# Use of Hemadsorption in a Case of Pediatric Toxic Shock Syndrome

**DOI:** 10.1155/2017/3818407

**Published:** 2017-07-16

**Authors:** Andrea Berkes, Edit Szikszay, János Kappelmayer, Adrienne Kerényi, Tamás Szabó, László Ujhelyi, Krisztina Bari, György Balla, József Balla

**Affiliations:** ^1^Department of Pediatrics, University of Debrecen, Debrecen 4012, Hungary; ^2^Institute of Laboratory Medicine, University of Debrecen, Debrecen 4012, Hungary; ^3^Department of Medicine, Faculty of Medicine, University of Debrecen, Debrecen 4012, Hungary; ^4^Fresenius Medical Care, NephroCare University of Debrecen, Debrecen 4012, Hungary; ^5^HAS-UD Vascular Biology and Myocardial Pathophysiology Research Group, Hungarian Academy of Sciences, Debrecen 4012, Hungary

## Abstract

**Background:**

Toxic shock syndrome is a potentially fatal toxin-mediated disease. The role of toxins in this clinical entity made us hypothesize that extracorporeal blood purification with CytoSorb® could play a beneficial role in the clinical management of toxic shock syndrome. This case report describes the successful treatment of toxic shock syndrome using a combination of renal replacement therapy and hemadsorption in a pediatric patient.

**Case Presentation:**

A 5-year-old girl with Down's syndrome presented with an inflamed area surrounding an insect bite, signs of systemic inflammation, and multiple organ failure. As previous attempts of immune modulation therapy were unsuccessful, renal replacement therapy was supplemented by the cytokine absorber CytoSorb. Treatment using this combination was associated with a rapid and significant stabilization in the hemodynamic situation and a decrease in inflammatory mediators within hours after the initiation of therapy. The application of CytoSorb therapy was simple and safe.

**Conclusion:**

The use of extracorporeal blood purification with CytoSorb proved potentially beneficial by removing toxins and inflammatory mediators in this case and could therefore play a role in the clinical management of toxic shock syndrome. Whether CytoSorb has the potential to even positively influence mortality in patients with toxic shock syndrome still needs to be confirmed.

## 1. Introduction

Toxic shock syndrome (TSS) is a potentially fatal toxin-mediated disease caused by infection from* Staphylococcus aureus* or Group A Streptococcus (GAS). This multisystem disorder is the consequence of an uncontrolled systemic inflammatory response syndrome caused by bacterial superantigens that broadly activate T-cells and lead to hypercytokinemia and hyperinflammation [1]. Consequently, the excessive release of various cytokines, leukotrienes, and prostaglandins, causing a “cytokine storm,” leads to the generation of the symptoms of this syndrome. The goal of therapy in TSS is to eradicate infection, mitigate the overwhelming inflammatory response, and prevent complications. Therefore, the fatal role of toxins in this disease entity made us hypothesize that extracorporeal blood purification with a novel sorbent, CytoSorb, might be capable of removing toxins, cytokines, and other inflammatory mediators and could play a beneficial role in the clinical management of TSS. This case report describes the successful treatment of toxic shock syndrome using CytoSorb in a five-year-old girl with Down's syndrome. Consent on publishing has been gathered from the patients' legal parents.

## 2. Case Report

A 5-year-old girl with Down's syndrome presented with an erythematous, indurated area on her right leg surrounding an insect bite noticed two days prior to admission. Oral cefaclor therapy was started for suspected systemic infection by* S. pyogenes* or* S. aureus/epidermidis* the day before admission by the GP. Despite antibiotics, the erythema continued to migrate proximally towards the inguinal region. On arrival at the emergency department, the conscious patient presented with diarrhea but stable vital signs: breathing rate 26 breaths/min, heart rate 110 beats/min, and blood pressure 98/57 mmHg. Blood examination revealed leukocytosis (15.44 G/L), neutrophilia (84.8%, absolute or 13.1 G/L), mild anemia (Hb 107 g/L, Hct 32%), a normal platelet count (238 G/L), and an elevated CRP (260.98 mg/L).

During the first two hours her status deteriorated so rapidly that she needed to be transferred to the intensive care unit. Capillary blood gas analysis revealed a severe uncompensated metabolic acidosis: pH 6.77, pCO_2_ 37.6 mmHg, HCO_3_ 5.2 mmol/L, and lactate 7 mmol/L. Septic shock was diagnosed and three intravenous fluid boluses (20 mL/kg) and oxygen therapy were started. Blood cultures were taken and the antibiotic therapy augmented by parenteral clindamycin and vancomycin. Due to an unsatisfactory cardiovascular response to fluid resuscitation, dopamine was started at 5 *μ*g/kg/min and then increased to 10 *μ*g/kg/min. Low ventricular contractility on ultrasonography required additional dobutamine at 5 *μ*g/kg/min while clinical signs of decreased systemic vascular resistance necessitated noradrenaline at 0.1 *μ*g/kg/min which had to be increased up to 0.5 *μ*g/kg/min later. Due to pronounced cardiovascular instability and suspected adrenal gland insufficiency, hydrocortisone was prescribed at 2 mg/kg/12 hours.

Considering the well-known immunodeficiency of patients with Down's syndrome, an antimycotic agent (fluconazole) was administered preventively, and intravenous immunoglobulin therapy (1 g/kg) for septic shock due to* S. pyogenes* or* S. aureus* was given on the day of admission and the following day. Fresh frozen plasma (15 mL/kg) and low molecular weight heparin (enoxaparin 6 mg/kg) were administered for laboratory signs of disseminated intravascular coagulation (DIC). Deep vein thrombosis of the right lower extremity was ruled out by Doppler ultrasonography. Chest X-ray revealed increased perihilar lung markings.

Eight hours after admission to the ICU an altered state of consciousness, hypercapnia, tachydyspnoe, and increasing oxygen needs required intubation and pressure controlled SIMV at PIP 17 cm H_2_O, PEEP 5 cm H_2_O, respiratory rate 25/min, and FiO_2_ 0.4.

Based on the Surviving Sepsis Campaign guidelines multiorgan failure was diagnosed. The patient had cardiovascular instability requiring complex vasoactive therapy (dopamine, dobutamine, noradrenaline, and milrinone); DIC indicated deteriorating liver function, poor lung function on ventilatory support alluding to Acute Respiratory Distress Syndrome (ARDS), and deteriorating kidney function. Microbiological examinations were inconclusive as to the causative pathogen.

**Figure 1:**
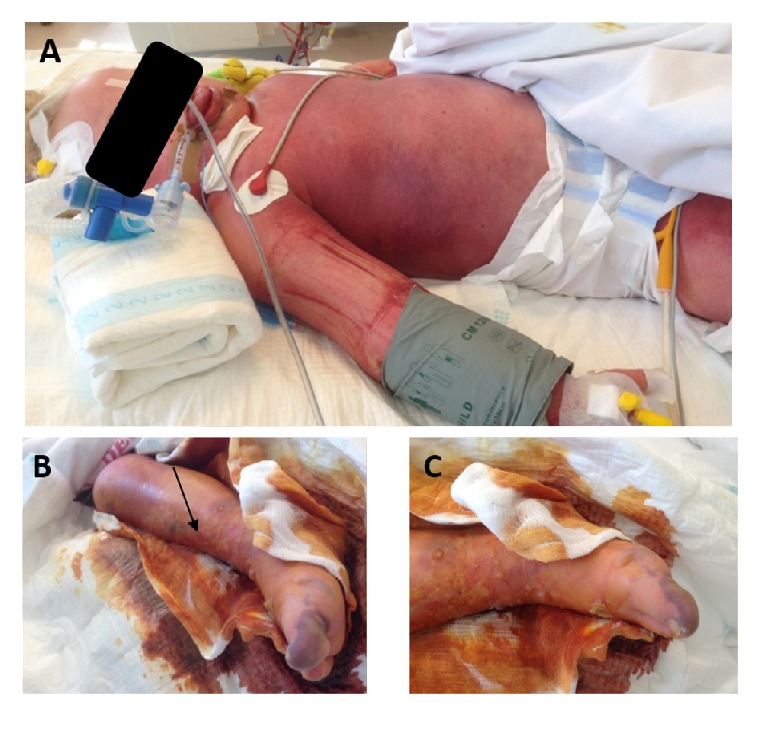
Diffuse erythroderma, purpura, and petechiae with severe generalized edema (A). Erythroderma, livid swelling, confluent bullae, and diffuse desquamation of the right leg (B, C). Arrow indicates site of the initial insect bite.

During the first 48 hours of therapy diffuse erythroderma with purpura and petechiae appeared, while the right leg became livid, and bullae appeared in places subjected to external pressure (Figure 1). Periodic pediatric surgery consultation excluded necrotizing fasciitis. The clinical picture now matched that of TSS. While skin lesions improved gradually and necrotic areas recovered fully under local conservative therapy from day seven onwards, new bullous eruptions continued to appear.

In an attempt to modulate the dysregulated immune response, plasma exchange therapy was performed without complications. Despite an increasing urine output during the first 24 hours from 0.4 to 5.4 ml/kg/hour her fluid balance remained positive. Thereafter, diuresis declined and the patient became anuric by hour 56 such that continuous venovenous hemodialysis was started. Thrombocytopenia, anemia, and hypoproteinemia were supported by blood products and an augmentation of the low neutrophil levels by granulocyte-colony stimulating factor tried on days four and five.

**Figure 2:**
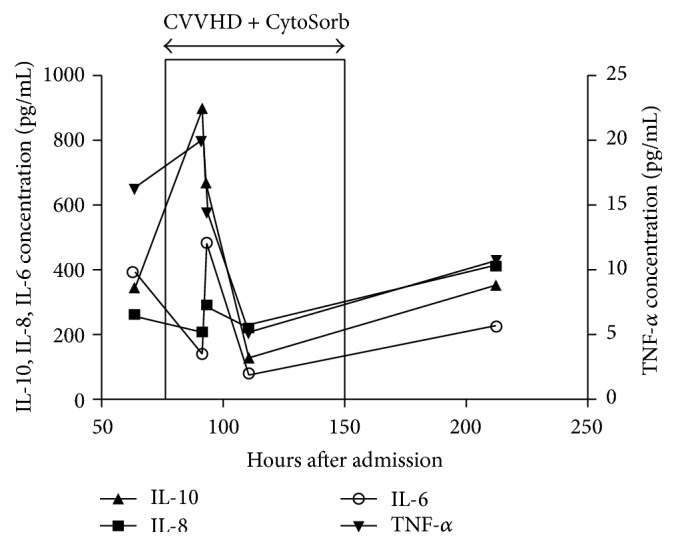
Course of proinflammatory and immunomodulatory cytokines before, during, and after combined CVVHD + CytoSorb treatment.

As previous attempts of immune modulation therapy were unsuccessful, renal replacement therapy was supplemented by the cytokine absorber CytoSorb (Cytosorbents Corp., Monmouth Junction, NJ, USA). CVVHD was performed with multiFiltrate (Fresenius Medical Care) machine, using a AV400S polysulfone dialyzer. Blood flow (*Q*b) of 40 mL/min could be achieved; dialysis fluid (MultiBic) flow (*Q*d) of 800 mL/hour was maintained, with an ultrafiltration rate of 100 mL/hour. Because of hepatic failure with DIC, regional citrate anticoagulation was used during the whole course of CRRT without any remarkable complication. As a result of a 72-hour uneventful course of the CVVHD-CytoSorb therapy, cardiovascular stability improved on lower vasopressor doses; FiO_2_ decreased from 0.95 to 0.4, airway pressures PIP/PEEP from 27/10 to 24/8 cm H_2_O, and respiratory rate from 25 to 15/min, respectively. In addition, levels of important laboratory parameters decreased (Table 1) as did plasma concentrations of proinflammatory and immunomodulatory cytokines (Figure 2). This attenuation of the inflammatory response correlated with the clinical improvement. The need for platelets decreased dramatically 48 hours after CytoSorb therapy and the diffuse erythroderma, purpura, and petechiae started to fade. CVVHD was continued for a further 8 days due to pronounced fluid overload, reaching normal rates of diuresis on day 17, three days after successful extubation and termination of the initial empiric antibiotic combination therapy. After six days of total parenteral nutrition the patient was switched to enteral feeding.

On day 20, her condition deteriorated again with tachypnea and desaturation together with elevated levels of CRP, PCT. This time, blood cultures revealed a staphylococcal infection; however, sepsis did not develop during the targeted antibiotic therapy. After 25 days the patient left the intensive care unit although her right ankle was still swollen.

The reappearance of fever accompanied by mildly elevated inflammatory laboratory parameters gave rise to the thought of deep tissue infection. MRI could not rule out an osteomyelitis of the os naviculare. The patient remained afebrile while the inflammation of the ankle became smaller under local conservative therapy. Complex physiotherapy tried to counteract muscle wasting and decreased tone. The girl was discharged home after 42 days where she recovered completely.

## 3. Discussion

In the present case report, we treated a 5-year-old girl with Down's syndrome who developed toxic shock syndrome and multiple organ failure after an insect bite, using a combination of CVVHD and hemadsorption. Treatment was associated with a rapid and significant stabilization of the hemodynamic situation accompanied by declining catecholamine dosage, improved respiratory status, and a decrease in inflammatory mediators within hours after initiation of therapy. Moreover, the need for platelets decreased dramatically and the diffuse erythroderma, purpura, and petechiae vanished.

These findings are in line with two published case reports on the use of CytoSorb in patients with toxic shock syndrome showing the potential role of this therapy in stabilizing hemodynamics, decreasing vasopressor requirements, improving lactate clearance, and reducing erythema as short term effects [2, 3].

The clinical picture in our case was ambiguous. The cutaneous lesion, as probable origin of the disease, suggested* Streptococcus pyogenes* as the causative agent, while the generalized erythroderma was typical for* Staphylococcus* infection. As bacteria from neither blood cultures nor skin and mucosal lesions were isolated, the microbiological background of our case remains unknown. Interestingly, a study that set out to quantify the ability of the CytoSorb polymer to adsorb a broad selection of inflammatory pathogen-associated molecular pattern molecules (PAMPs), damage-associated molecular pattern molecules (DAMPs), and cytokines from whole blood in a single compartment in vitro recirculation system provided that substantial quantities of a broad spectrum of DAMPS, PAMPS, and cytokines (i.e., S100A8, complement C5a, procalcitonin, HMGB-1, MIP1-*α*, IL-6, IFN-*γ*, TNF-*α*, Staph enterotoxin TSST-1, and aflatoxin B1) were removed [4]. Therefore, direct removal of streptococci toxins may potentially have played a role in the current case. These results might be of particular interest in a case such as the one presented herein, where the pathological origin is not clear but the diagnosis of TSS was established.

One of the striking features of systemic TSS is the speed of excess cytokine and toxin production. Our patient had been treated with multiple immune-modulatory therapies (i.e., hydrocortisone, IVIG, and plasma exchange therapy) achieving only moderate reductions in the laboratory parameters that characterize systemic inflammation. However, from the start of CVVHD/CytoSorb initiation, plasma levels of inflammatory mediators such as IL-6, IL-10, IFN-*γ*, CRP, and PCT but also of metabolic parameters (i.e., lactate) started to decrease rapidly. This attenuation of the inflammatory response correlated well with the clinical improvement of the patient.

Previous studies have shown that inflammatory mediators can be removed from the circulation with different blood purification techniques such as plasma exchange, high-volume and very high-volume hemofiltration, and a number of hybrid therapies encompassing high-permeability hemofiltration, super high-flux hemofiltration, hemadsorption or coupled filtration, and adsorption [5]. These therapeutic modalities have been associated with lower mortality in patients with sepsis [6, 7]. Whether CytoSorb has the potential to positively influence mortality in TSS patients needs to be confirmed in randomized, controlled trials.

However, despite the fact that most experience with CytoSorb in the clinical setting to date is still limited to case reports, the first multicenter randomized controlled study using treatment with CytoSorb hemadsorption in septic patients with acute lung injury showed that application of the device over a 7-day treatment period was safe and effectively decreased the blood concentration levels of several key cytokines [8–11]. From this perspective, further studies to elucidate the potential clinical impact of this new therapy option are urgently warranted.

## 4. Conclusion

The use of extracorporeal blood purification with a novel sorbent, CytoSorb, proved potentially beneficial by removing toxins, cytokines, and other inflammatory mediators in this case of toxic shock syndrome in a pediatric patient and could therefore play a future role in the clinical management of toxic shock syndrome. Whether CytoSorb has the potential to even positively influence mortality in patients with toxic shock syndrome needs to be confirmed in randomized, controlled trials.

## Figures and Tables

**Table 1:** Laboratory parameters during the course of multiple immune-modulatory therapies including CVVHD + CytoSorb.

Time from admission	pH	Lactate [mmol/L]	CRP [mg/L]	PCT [*μ*g/L]	WBC [10^3^/*μ*L]	Platelets [10^3^/*μ*L]	BUN [mmol/L]	Creatinine [*μ*mol/L]	ALT [U/L]	AST [U/L]	LDH [U/L]
0	6,42	8.6	178.7	218.4	14.97	204	10.5	196	38	21	344
6 hr	Start of hydrocortisone, IVIG										
15 hr	7.35	4.4	114	95.07	8.67	67	11.1	134	—	—	—
42 hr	7.37	3.9	105.1	32.85	7.72	34	17.3	147	—	—	—
63 hr	7.39	3.6	113.5	20.54	8.15	12	24.3	210	—	—	—
67 hr	Start of plasma exchange therapy										
69 hr	7.39	4.2	87.46	19.02	6.96	21	26.4	223	270	150	887
78 hr	Start of CVVHD-CytoSorb										
91 hr	7.41	1.9	50.5	4.06	1.91	5	18.8	163	214	101	554
133 hr	7.47	1.5	87.46	3.33	4.31	5	12.8	98	274	146	676
150 hr	End of CVVHD-CytoSorb										
212 hr	7.39	0.7	76.01	2.8	3.78	20	7.6	78	78	94	587

CRP: C-reactive protein, PCT: procalcitonin, WBC: white blood cell, BUN: blood urea nitrogen, ALT: alanine aminotransferase, AST: aspartate aminotransferase, LDH: lactate dehydrogenase, IVIG: intravenous immune globulin, and CVVHD: continuous venovenous hemodialysis. Normal values of laboratory results provided by the examiner institution: lactate: 0,5–2,2 mmol/L, CRP: <2,2 mg/L, PCT: 0,5 *μ*g/L, WBC: 4,5–11,5 G/L, platelets: 150–500 G/L, BUN: 1,8–6,4 mmol/L, creatinine: 18–53 mmol/L, ALT: <40 U/L, AST: <40 U/L, and LDH: <340 U/L.

## Abbreviations

TSS: Toxic shock syndrome
GAS: Group A Streptococcus
Hb: Hemoglobin
Hct: Hematocrit
CRP: C-reactive protein
DIC: Disseminated intravascular coagulation
PIP: Peak inspiratory pressure
PEEP: Positive end-expiratory pressure
PCT: Procalcitonin
WBC: White blood cell
IVIG: Intravenous immune globulin
CVVHD: Continuous venovenous hemodialysis.
